# Vitamin D in Peri-Implant and Periodontal Tissue

**DOI:** 10.3390/dj13100448

**Published:** 2025-09-30

**Authors:** Felipe de Souza Duarte, Nathália Dantas Duarte, Gabriel Mulinari-Santos, Paula Buzo Frigério, Roberta Okamoto, Rogerio Leone Buchaim, Daniela Vieira Buchaim, João Paulo Mardegan Issa

**Affiliations:** 1 Department of Diagnosis and Surgery, Araçatuba School of Dentistry (FOA-UNESP), São Paulo State University, Araçatuba 16015-050, Brazil; fs.duarte@unesp.br (F.d.S.D.); nd.duarte@unesp.br (N.D.D.); paula.frigerio@unesp.br (P.B.F.); 2 Department of Basic Sciences, Araçatuba School of Dentistry (FOA-UNESP), São Paulo State University, Araçatuba 16015-050, Brazil; gabriel.mulinari@unesp.br (G.M.-S.); roberta.okamoto@unesp.br (R.O.); 3 Department of Biological Sciences, Bauru School of Dentistry (FOB-USP), University of São Paulo, Bauru 17012-901, Brazil; rogerio@fob.usp.br; 4 Graduate Program in Anatomy of Domestic and Wild Animals, School of Veterinary Medicine and Animal Sciences, University of São Paulo (FMVZ-USP), São Paulo 05508-270, Brazil; danibuchaim@alumni.usp.br; 5 Medical School, University Center of Adamantina (FAI), Adamantina 17800-000, Brazil; 6 Department of Postgraduate, Dentistry School, Faculty of the Midwest Paulista (FACOP), Piratininga 17499-010, Brazil; 7 Department of Basic and Oral Biology, Ribeirão Preto School of Dentistry (FORP-USP), University of São Paulo, Ribeirão Preto 14040-904, Brazil

**Keywords:** bone regeneration, calcitriol, dentistry, osseointegration, peri-implantitis, periodontal diseases, review, vitamin D

## Abstract

This review aims to provide an overview of the role of vitamin D in peri-implant and periodontal tissue. Electronic searches were carried out of the PubMed/Medline database. Since this is a narrative review, no systematic search, meta-analysis, or statistical analysis was performed. Vitamin D plays a crucial role in bone balance and metabolism, contributing to reducing early implant failure and improving dental implant osseointegration. Vitamin D deficiency poses a challenge to clinical outcomes, and its supplementation can be an effective alternative to overcome this limitation. The results reported in this article show that vitamin D application on implants can improve the osseointegration, bone-to-implant contact, implant stability, and bone density. Moreover, vitamin D supplementation can increase RUNX2, ALP, OPN, and OCN expression, contributing to periodontal tissue health and its regeneration. Together, findings provide an overview of these topics and present future perspectives for clinical practice in dentistry.

## 1. Introduction

Vitamin D has been increasingly explored in dentistry as a biological adjuvant to enhance bone regeneration across various clinical situations since it plays a pivotal role in bone metabolism, particularly in regulating bone turnover and maintaining adequate bone mineral density [1,2]. Among these, implant dentistry has received significant attention, as adequate serum levels of vitamin D have been associated with improved osseointegration and reduced risk of early implant failure, especially in patients with low bone density or systemic conditions that compromise bone healing such as osteoporosis, diabetes mellitus, or cardiovascular diseases [1,2]. Vitamin D is naturally obtained from the diet or synthesis in the skin and is converted to an active hormone known as calcitriol, which is responsible for promoting intestinal absorption of calcium and phosphate [2]. Calcitriol is able to support bone remodeling and mineral homeostasis [2,3]. In addition to systemic supplementation, evidence supports the localized delivery of vitamin D through implant surface coatings, hydrogels, or scaffold incorporation, aiming to promote peri-implant bone formation and accelerate healing in critical-size defects [3,4].

Given that vitamin D deficiency affects more than one billion individuals globally and costs more than USD 1.56 billion in the United States of America alone [5,6], its optimization through supplementation has become a relevant topic in translational dental research. Traditionally administered systemically, vitamin D is now being investigated for innovative local applications in dentistry, such as its incorporation into bone graft materials, implant coatings, or biodegradable carriers to enhance peri-implant bone healing [3,4]. These strategies aim to overcome systemic limitations like variable absorption and patient compliance, providing targeted and sustained release at the surgical site [7,8].

In addition, vitamin D is studied in the management of periodontal disease, where it exerts anti-inflammatory and immunomodulatory effects [9]. Both systemic and local applications may support periodontal regeneration when combined with bone grafts, barrier membranes, or biologic mediators. Furthermore, patients with osteoporosis, diabetes, or chronic inflammatory conditions may benefit significantly from vitamin D supplementation, which has the potential to improve surgical outcomes and optimize tissue response to implant or regenerative therapies [10,11,12].

These therapeutic benefits are due to the biological role of vitamin D in bone metabolism. It is a prohormone synthesized in the skin upon exposure to ultraviolet B radiation or obtained from dietary sources such as oily fish, including salmon, tuna, and sardines [13,14]. Once produced, vitamin D undergoes hepatic conversion to 25-hydroxyvitamin D, followed by renal hydroxylation into its active form, calcitriol [15,16]. This active form of vitamin D found in the body regulates bone turnover by modulating the activity of osteoblasts and osteoclasts as well as by promoting the expression of genes involved in bone mineralization [17,18,19]. Through these mechanisms, vitamin D contributes to calcium and phosphorus homeostasis as well as supports overall skeletal integrity.

**Figure 1:**
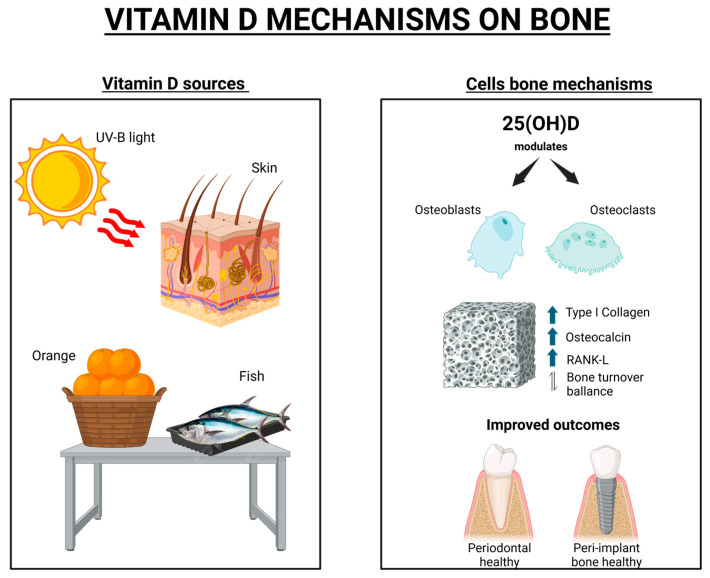
Schematic representation of vitamin D mechanisms on bone tissue, including its sources and cellular actions involved in bone metabolism, created with BioRender.com. Available in: https://app.biorender.com/illustrations/689629c9ef5083a9587583cf (accessed on 2 September 2025).

Additionally, vitamin D supplementation may influence the therapeutic outcomes of patients, particularly with systemic conditions, in dentistry [20,21,22]. Therefore, this narrative review aims to critically examine the current evidence regarding systemic supplementation and local vitamin D in dental bone regeneration, focusing on animal and clinical studies. This review provides an important overview of studies and scientific evidence that aims to help clinical professionals understand this theme. Special emphasis is placed on its role in peri-implant repair, periodontal regeneration, alveolar bone healing, and the management of periodontal tissues and disease as well as on innovative strategies for translation into clinical application, as illustrated in Figure 1.

This narrative review was conducted following the guidelines of the Scale for the Assessment of Narrative Review Articles (SANRA) [23]. Electronic searches were carried out of the PubMed/Medline database. The search included the following keywords: peri-implant, periodontal regeneration, periodontal disease, management of periodontal disease, vitamin D, and related terms described in this review, with no time or language restrictions. The collected information was from in vitro, in vivo, and clinical studies according to items 1 and 5 of the SANRA methodology. Since this is a narrative review, no systematic search, meta-analysis, or statistical analysis was performed.

## 2. Role of Vitamin D in Peri-Implant Tissue

**Figure 2:**
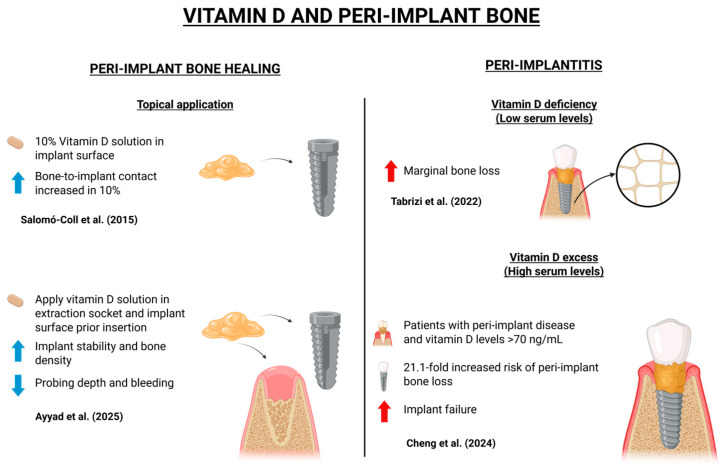
Schematic illustration showing the correlation between vitamin D and peri-implant bone, created with BioRender.com. Available in: https://app.biorender.com/illustrations/689645651ab812100f4c4eff?slideId=f59c58ff-e69d-413f-9260-3c67687989b6 (accessed on 2 September 2025) [2,3,4,28].

It is well-established that vitamin D, also known as calcitriol, plays a crucial role in peri-implant bone homeostasis and implant osseointegration [14,15]. Vitamin D is primarily obtained through sunlight exposure and diet, and its supplementation is essential for preventing hypovitaminosis D, mainly because vitamin D deficiency has been associated with impaired peri-implant bone healing [7,17]. The correlation between vitamin D and peri-implant bone is illustrated in the schematic diagram in Figure 2, while Table 1 presents the content analysis of the selected articles on this topic.

**Table 1:** Details of the selected articles on the role of vitamin D on peri-implant bone. Abbreviations: BIC—bone-to-implant contact; DMSO—dimethyl sulfoxide; μg/kg/day—micrograms per kilogram per day; IU—international unit; µL—microliter; µg/mL—micrograms per milliliter; IU/kg—IU per kilogram; IU/day—IU per day.

Authors and Year	Study Type	Population (*N*), Sex and Age	Details of Vitamin D	Conclusion
Wu et al. (2013) [24]	In vivo	Wistar male rats *N* = 30 Age (weeks) = 10–11	Vitamin D3 12 μg/kg/day via gavage for 14 days (started 3 days after implant surgery). Co-intervention with insulin subcutaneously twice daily (5.5 IU at 8 p.m., 3.5 IU at 8:00 a.m.).	Both insulin and vitamin D3 alone improved some outcomes such as implant fixation in diabetic rats, but combination therapy had superior effects.
Gomes-Ferreira et al. (2023) [25]	In vivo	Wistar male rats *N* = 24 Age not reported	Vitamin D 0.1 µg/kg/day via gavage, for 30 days. Co-intervention with daily subcutaneous teriparatide 0.5 µg/kg/day.	Vitamin D combined with teriparatide in orchiectomized rats with induced osteoporosis significantly increased bone volume and improved bone quality around tibial implants.
Pitol-Palin et al. (2025) [26]	In vivo	Wistar male rats *N* = 15 Age (months) = 3	Dip-coating of titanium implants with vitamin D3 (Addera D3^®^). Concentrations tested: vD40 µL: 40 µL vitamin D3 in 100 mL DMSO; vD400 µL: 400 µL vitamin D3 in 100 mL DMSO.	The vD400 µL concentration led to improved bone microarchitecture.
Salomó-Coll et al. (2016) [3]	In vivo	Dogs (American Foxhound) *N* = 6 Sex not specified Age (years) = 1	Implant submerged in 10% vitamin D_2_ (ergocalciferol) 10% solution.	Topical application of vitamin D on immediate implants did not significantly enhance osseointegration compared to controls. However, vitamin-D-treated implants showed less crestal bone loss and about 10% higher BIC contact after 12 weeks.
Ayyad et al. (2025) [4]	Randomized controlled trial	Healthy ASA I *N* = 24 Sex not specified Age (years): 21–40 years	Topical vitamin D_3_ (calcitriol) 1 µg/mL emulgel applied to both the implant surface and osteotomy site immediately before implant placement.	Topical vitamin D_3_ improved peri-implant soft tissue healing (reduced probing depth and bleeding index), decreased postoperative pain, and enhanced implant stability and bone density over 6 months.
Dvorak et al. (2012) [27]	In vivo	Ovariectomized female rats *N* = 48 Age (months) = 3	Vitamin D was provided via diet containing 1000 IU vitamin D_3_ per kg of feed.	Dietary vitamin D significantly improved BIC and peri-implant bone density in ovariectomized rats.
Cheng et al. (2024) [2]	Retrospective case-control	Wistar male rats *N* = 24 Age (weeks) = 8	Vitamin D_3_ supplementation of 2500 IU/kg diet for 6 weeks prior to surgery and continuing until sacrifice.	Vitamin D supplementation improved osseointegration, as evidenced by increased BIC and higher removal torque values compared to the control group.
Tabrizi et al. (2022) [28]	Prospective cohort	Healthy *N* = 40 18 males and 22 females Age (years) = 18–50	Oral vitamin D_3_ capsules of 1000 IU/day, starting 6 weeks before surgery and continuing for 3 months after implant placement.	Oral vitamin D_3_ supplementation improved early osseointegration, showing higher implant stability quotient values at 6 and 12 weeks compared to placebo.

### 2.1. Peri-Implant Bone Healing

Regarding implant osseointegration, systemic conditions such as diabetes mellitus can hinder peri-implant healing. Vitamin D supplementation, alone or in combination with other agents, has been investigated as a strategy to improve osseointegration. Wu et al. (2013) demonstrated that vitamin D3 combined with insulin normalized glycemic levels in diabetic rats and enhanced bone volume and osseointegration percentages [24].

However, vitamin D3 alone did not produce statistically significant improvements. Additionally, Gomes-Ferreira et al. (2023) reported that the combination of teriparatide (a parathyroid hormone analog used in osteoporosis treatment) with vitamin D improved peri-implant bone quality and increased bone volume in ovariectomized rats [25]. A recent study by Pitol-Palin et al. (2025) showed that the functionalization of titanium implant surfaces with Vitamin D can promote bone mineralization, cell viability, and cell interaction in a rat model [26].

Topical application of vitamin D on implant surfaces has also shown promise. In an animal model, Salomó-Coll et al. (2016) submerged implants in a 10% vitamin D solution and found a 10% increase in bone-to-implant contact (BIC) after 12 weeks compared to controls [3]. Supporting these findings, Ayyad et al. (2025) demonstrated that applying vitamin D to the extraction socket and implant surface before insertion increased implant stability and bone density while reducing probing depth and bleeding indices over a 6-month follow-up [4]. Moreover, an animal study with ovariectomized rats, conducted by Dvorak et al. (2012), demonstrated that vitamin D deficiency resulted in reduced BIC and impaired cortical bone formation. Rats receiving daily vitamin D supplementation (2400 IU/kg for 8 weeks) exhibited significantly improved peri-implant bone parameters compared to the control group [27]. All the above findings represent results of animal studies, and human/clinical studies need to be carried out to explore, confirm, or deny these findings.

### 2.2. Peri-Implantitis

Vitamin D excess may also negatively impact dental implant outcomes, according to a recent study by Cheng et al. (2024), which demonstrated that patients with peri-implant disease and serum vitamin D levels exceeding 70 ng/mL, classified as hypervitaminosis D, had a 21.1-fold increased risk of peri-implant bone loss compared to patients with intermediate serum levels. Moreover, implant failure was more frequent in this group. The deleterious effects of hypervitaminosis D were more pronounced in maxillary implants compared to mandibular ones, as demonstrated in this research [2].

Marginal bone loss (MBL) has been shown to correlate with serum vitamin D levels. In a prospective cohort study, Tabrizi et al. (2022) analyzed 90 patients who received implants in the first or second molar regions, dividing them into three groups based on serum vitamin D concentrations: deficient, insufficient, and sufficient. After 12 months of follow-up, group 3 (sufficient vitamin D) exhibited the lowest mean MBL, suggesting that low serum vitamin D may contribute to greater bone loss [28].

Corroborating these findings, Mahendra et al. (2025) demonstrated, in a five-year retrospective study, that systemic conditions as diabetes and vitamin D deficiency are associated with a lower implant survival rate, in addition to factors like age, smoking status, and prosthetic design [29].

## 3. Role of Vitamin D in Periodontal Disease

Vitamin D deficiency levels have been significantly linked to periodontal disease. In a case–control study developed by Laky et al. (2017), patients with periodontitis had serum 25(OH)D levels below 50 ng/mL, suggesting that hypovitaminosis D may contribute to disease progression [30]. Contributing to these findings, Yildirim et al. (2025) analyzed gingival crevicular fluid from 200 patients categorized by serum vitamin D levels (<10 ng/mL vs. ≥10 ng/mL). Elevated levels of matrix metalloproteinase-9 (MMP-9) were observed in the vitamin-D-deficient group, suggesting a correlation between hypovitaminosis D and periodontitis severity [31].

The periodontal ligament (PDL) contains diverse cells, including fibroblasts, cementoblasts, osteoblasts, and osteoclasts, which contribute to tissue maintenance and regeneration [16,18]. Nebel et al. (2015) explored the effects of 30 ng/mL vitamin D on PDL cells. While no changes were observed in morphology or cell count at 24 h, there was an upregulation of osteopontin and osteocalcin mRNA. At 48 h, vitamin D treatment enhanced alkaline phosphatase activity, indicating a positive osteogenic effect [32]. Similarly, Pereira et al. (2024) showed that treatment of PDL cells with 1,25(OH)_2_D_3_ enhanced osteoblastic potential without affecting viability, significantly upregulating the expression of RUNX2, ALP, and OCNP, and increasing mineral nodule formation in vitro [33]. Vitamin D also demonstrates anti-inflammatory properties in periodontal disease. In a study by Han et al. (2019), rats with periodontal disease received intraperitoneal injections of 25-hydroxyvitamin D3 for 8 weeks. The treatment reduced serum RANKL, IL-1, and TNF-α levels and limited alveolar bone loss [34].

**Figure 3:**
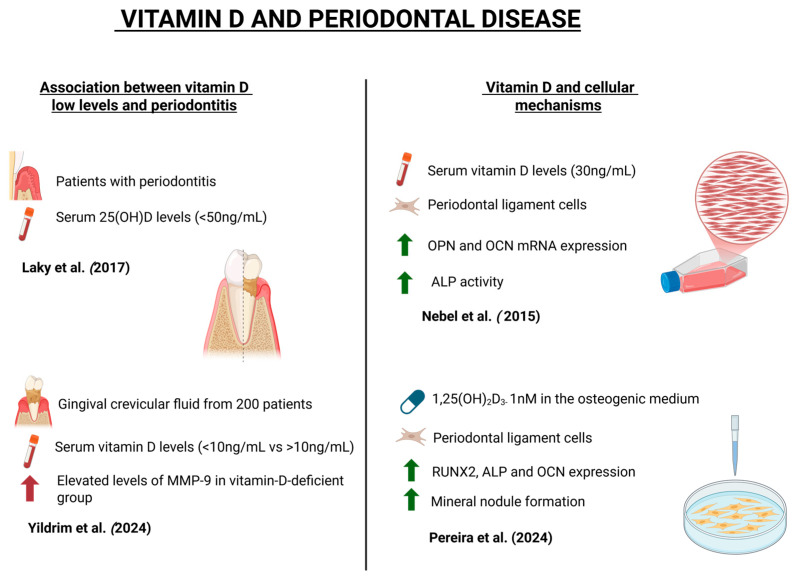
Schematic illustration demonstrating the mechanisms involved in the role of vitamin D and periodontal disease, created with BioRender.com. Available in: https://app.biorender.com/illustrations/689a194146accc852fa8fe50 (accessed on 2 September 2025) [30,31,32,33].

Vitamin D supplementation has therapeutic potential in managing periodontal disease, particularly in patients with systemic conditions such as diabetes mellitus. RamaPrabha et al. (2023) investigated the effects of weekly oral vitamin D supplementation (60,000 IU for 8 weeks) in patients with type II diabetes mellitus and generalized chronic periodontitis [1]. Clinical parameters—including plaque index, gingival bleeding, probing depth, and attachment levels—showed significant improvement in the vitamin-D-treated group, indicating an immunomodulatory effect. The role of vitamin D and periodontal disease is illustrated in the schematic diagram in Figure 3, while Table 2 presents the content analysis of the selected articles on this topic.

## 4. Overview of Concepts and Findings

Vitamin D is a prohormone synthesized in the skin following exposure to ultraviolet B (UVB) radiation or obtained through dietary sources such as oily fish, including salmon, tuna, and sardines [12,13]. Cutaneous synthesis begins with the conversion of 7-dehydrocholesterol to previtamin D upon UVB exposure, which is then thermally isomerized into vitamin D [3,7,10]. In the liver, vitamin D3 is hydroxylated into 25-hydroxyvitamin D [25(OH)D], also known as calcidiol, and subsequently converted in the kidneys into its biologically active form, calcitriol [14,15,16].

It also plays a pivotal role in bone metabolism, particularly in regulating bone turnover and maintaining adequate bone mineral density [14,17]. Its supplementation is essential for the prevention and management of bone-related disorders such as osteoporosis and osteopenia [5,14]. Calcitriol enhances the expression of key proteins involved in bone mineralization, including those that regulate calcium and phosphorus homeostasis [12,17]. Moreover, it modulates osteoblast and osteoclast activity, thereby influencing the dynamic equilibrium between bone formation and resorption [16,18].

Based on the findings of the articles included in this work, we suggest that vitamin D deficiency is involved in impaired peri-implant bone healing [24,25,26,27,28,30,31,32,33,34]. Low serum levels of vitamin D in the blood are associated with lower implant survival [28], increased marginal bone loss [30], a reduced BIC, and less cortical bone formation [34]. Similarly, low levels of vitamin D can also be associated with periodontal disease progression [31].

The systemic effects of vitamin D supplementation are influenced by dosage, typically expressed in international units (IU), treatment frequency, and individual physiological conditions. Special populations such as pregnant women [19], postmenopausal individuals [20,21], and patients with chronic conditions such as diabetes [10], osteoporosis [11], hypertension [22], or cardiovascular disease [9] may exhibit varying responses to supplementation, as demonstrated by a consensus of the above studies.

The reviewed articles suggest that a solution to overcome this clinical situation is supplementation with vitamin D, alone or in association with other substances, indicated by health professionals like doctors or dentists. Teriparatide, as demonstrated by Gomes-Ferreira et al. (2023) [25], when combined with vitamin D, increases bone volume and enhances bone quality in orchiectomized rats. In diabetic rats, vitamin D associated with insulin showed enhanced bone volume and osseointegration percentages [24].

An anti-inflammatory effect is associated with vitamin D treatments, reducing serum levels of RANKL, TNF-α, and IL-1 and lowering alveolar bone loss in animals with periodontitis [3]. From a cellular perspective, treatment with 1,25(OH)2D3 in low-osteoblastic-potential cells was able to upregulate gene expression of RUNX2, ALP, and OCN [33]. In human periodontal ligament (PDL) cells, vitamin D attenuated the expression of IL-6 and CXCL1, supporting bone regeneration and mitigating inflammatory processes [32].

This review has some limitations, including the heterogeneity of the study designs, supplementation protocols, and baseline vitamin D levels, as well as the predominance of preclinical studies, which may limit direct clinical applicability of the findings. Another limitation is that only one database was searched. The absence of standardized outcome measures also hinders the definition of optimal dosages. Therefore, future studies should prioritize large-scale randomized clinical trials to establish evidence-based supplementation protocols for implant therapy, investigate long-term outcomes, assess combinations with other anabolic or antiresorptive agents, and explore genetic factors influencing individual responses may clarify the role of vitamin D in peri-implant bone metabolism.

## 5. Conclusions

Vitamin D plays a crucial role in bone metabolism, being able to modulate bone cells and gene expression involved in bone balance. Vitamin D deficiency constitutes a challenge to clinical results, and supplementation through the diet or other means can help to improve dental implant osseointegration, bone healing, bone volume, and periodontal disease treatments, resulting in better clinical outcomes. Preventive supplementation with vitamin D can also be a good alternative to avoid the bone impact of this vitamin’s deficiency. Additionally, populations with specific physiological or pathological conditions may respond differently to vitamin D supplementation, emphasizing the need for individualized treatment strategies and more studies to confirm these findings.

## Figures and Tables

**Table 2:** Details of the selected articles about the role of vitamin D in periodontal disease. Abbreviations: 25(OH)D—25-hydroxyvitamin D; MMP-8—matrix metalloproteinase-8; PDL—periodontal ligament; COPD—chronic obstructive pulmonary disease; IU—international units; ng/mL—nanograms per milliliter; nmol/L—nanomoles per liter; M—molar unit.

Authors and Year	Study Type	Population (N), Sex and Age	Details of Vitamin D	Conclusion
Laky et al. (2017) [30]	Observational case–control	Periodontal disease N = 29 12 males and 17 females Mean age (years) = 35.41 ± 7.7 Healthy N = 29 8 males and 21 females Mean age (years) = 35.45 ± 7.4	Serum 25(OH)D levels were measured, but no supplementation was given. Vitamin D deficiency was defined as serum levels < 50 nmol/L.	Vitamin D deficiency (<50 nmol/L) was significantly associated with periodontal disease, with affected patients showing lower serum levels than healthy controls.
Yildirim et al. (2025) [31]	Observational comparative	Generalized stage III–IV periodontitis N = 63 38 females and 25 males Mean age (years) = 46.0 ± 8.0	Serum 25(OH)D levels were measured using ELISA. Vitamin D deficiency was defined as <20 ng/mL, insufficiency as 20–30 ng/mL, and sufficiency as >30 ng/mL.	Lower serum vitamin D levels were linked to higher MMP-8 activity, suggesting a role in increased periodontal tissue destruction.
Nebel et al. (2015) [32]	In vitro	Chronic periodontitis N = 12 Gingival tissue samples were collected from males and females Age (years) = 30–65	PDL cells were treated with 1α,25-dihydroxyvitamin D_3_ at concentrations of 10^−8^ M and 10^−7^ M.	Vitamin D_3_ stimulated bone-forming activity and suppressed inflammation in periodontal ligament cells.
Pereira et al. (2024) [33]	Observational cross-sectional	Osteoporosis N = 15 Mean age (years) = 57.3 ± 4.9 Healthy N = 15 Mean age (years) = 57.0 ± 4.1	Serum 25(OH)D levels were measured using electrochemiluminescence immunoassay. Deficiency was defined as <20 ng/mL, insufficiency as 20–29 ng/mL, and sufficiency as ≥30 ng/mL.	Lower vitamin D levels were associated with more severe periodontal attachment loss in postmenopausal women with osteoporosis.
Han et al. (2019) [34]	In vivo	Wistar male rats N = 40 Age (weeks) = 8	25(OH)D_3_ via intraperitoneal injection 5 μg/kg, 3 times per week, 12 weeks.	25(OH)D_3_ diminished bone loss, lung damage, and systemic inflammation in rats with periodontitis and/or COPD.
Ramaprabha et al. (2023) [1]	Randomized clinical trial	Non-diabetic male N = 46 Diabetic male N = 46 Age (years) = 35–60	Oral vitamin D3 granules, 60,000 IU once a week for 8 weeks after scaling and root planing.	Vitamin D supplementation alongside scaling and root planing significantly improved periodontal health and serum vitamin D levels, with greater benefits in patients without diabetes.
Mahendra et al. (2025) [29]	Retrospective cohort	N (patients) = 143 N (implants) = 161 Male = 54.7% Female = 45.3% Age (years) = 18–62	Past medical history included self-reported vitamin D deficiency (6.3% of participants). Vitamin D deficiency was assessed from patient records but was not significantly associated with implant survival.	Dental implant survival is high over five years, but success depends on systemic health, habits, and prosthetic factors, while vitamin D deficiency alone is not a predictor of failure.

## Data Availability

No new data were created or analyzed in this study. Data sharing is not applicable to this article.

## Abbreviations

VD3	Vitamin D3 (cholecalciferol)
BIC	Bone-to-Implant Contact
ITB	Interthread Bone
NBF	New Bone Formation
HLM	Histological Linear Measurement
BV	Bone Volume
BV/TV	Bone Volume/Tissue Volume
Tb.Th	Trabecular Thickness
Tb.N	Trabecular Number
Tb.Sp	Trabecular Separation
Po-tot	Total Porosity
MBL	Marginal Bone Loss
PDL	Periodontal Ligament
MMP-9	Matrix Metalloproteinase-9
GCF	Gingival Crevicular Fluid
PPD	Periodontal Probing Depth
CAL	Clinical Attachment Level
BOP	Bleeding on Probing
BMI	Body Mass Index
IL	Interleukin (IL-1, IL-6, IL-10)
TNF-α	Tumor Necrosis Factor Alpha
RANKL	Receptor Activator of Nuclear Factor Kappa-B Ligand
OCN	Osteocalcin
OPN	Osteopontin
ALP	Alkaline Phosphatase
RUNX2	Runt-Related Transcription Factor 2
ASPN	Asporin
BMP-2	Bone Morphogenetic Protein 2
VDR	Vitamin D Receptor
COPD	Chronic Obstructive Pulmonary Disease
PI	Plaque Index
GBI	Gingival Bleeding Index
SRP	Scaling and Root Planing
IU	International Units
UI/kg	International Units per Kilogram
